# Self‐reported autonomic dysfunction could be a predictive marker for sarcopenia in Parkinson's disease

**DOI:** 10.1111/ggi.70104

**Published:** 2025-06-24

**Authors:** Motohiro Okumura, Yohei Mukai, Reiko Saika, Yuji Takahashi

**Affiliations:** ^1^ Department of Neurology National Center Hospital, National Center of Neurology and Psychiatry Tokyo Japan

**Keywords:** aging, autonomic dysfunction, Parkinson's disease, sarcopenia, Scales for Outcomes in Parkinson's Disease‐Autonomic Questionnaire (SCOPA‐AUT)

## Abstract

**Aim:**

Autonomic dysfunction and motor symptoms are prevalent in Parkinson's disease (PD). Motor symptoms influence sarcopenia; however, the association between sarcopenia and non‐motor symptoms, particularly autonomic dysfunction, remains unclear. This study determined the effect of autonomic dysfunction on sarcopenia in patients with PD.

**Methods:**

Consecutive patients with PD (Hoehn and Yahr stages 1–3) without apparent dementia were screened. The Scales for Outcomes in Parkinson's Disease‐Autonomic Questionnaire (SCOPA‐AUT) was utilized to evaluate the severity of autonomic dysfunction. Sarcopenia was assessed using the 2019 Asian Diagnostic Criteria. This study examined whether the SCOPA‐AUT and its domains were associated with sarcopenia and used receiver operating characteristic analysis to evaluate their predictive performance.

**Results:**

Of the 124 patients (76 [61%] men; median age, 68 years) included, sarcopenia was identified in 31 (25%). Poisson regression analysis with a robust variance estimator showed that a higher SCOPA‐AUT score is associated with sarcopenia (prevalence ratio 1.078, 95% CI 1.034–1.122, *p* < 0.001). Regarding SCOPA‐AUT domains, higher scores for gastrointestinal functioning, urinary functioning and pupillomotor functioning were significantly associated with sarcopenia. Receiver operating characteristic analysis showed that the optimal cut‐off value for SCOPA‐AUT was 16 (area under the curve 0.730, 95% CI 0.615–0.844). For each SCOPA‐AUT domain, a cut‐off of 8 for gastrointestinal functioning (area under the curve 0.744, 95% CI 0.630–0.858) predicted sarcopenia more reliably than urinary and pupillomotor functioning.

**Conclusions:**

Higher SCOPA‐AUT scores, particularly in the gastrointestinal function domain, might be an optimal predictive marker for sarcopenia in patients with PD. **Geriatr Gerontol Int 2025; 25: 1074–1081**.

## Graphical abstract

Autonomic dysfunction, assessed by the Scales for Outcomes in Parkinson's Disease‐Autonomic Questionnaire, was significantly associated with sarcopenia in patients with Parkinson's disease. Among all autonomic domains, the gastrointestinal score had the strongest predictive ability for sarcopenia.

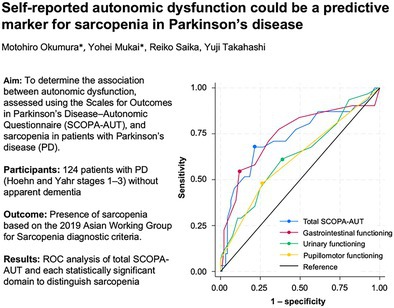

## Introduction

Sarcopenia is defined as a progressive quantitative and qualitative muscle impairment that increases with age.^1^ Aging is a well‐established risk factor for the onset of Parkinson's disease (PD). Consequently, sarcopenia might be comparatively common in approximately 30% of patients with PD compared with age‐matched healthy controls, and contribute to the progression of the PD stage and severity of motor dysfunction.^2^, ^3^ Therefore, elucidating the factors associated with sarcopenia and early intervention in patients with PD are crucial.

Studies have investigated the relationship between motor symptoms and sarcopenia in patients with PD, suggesting that longer disease duration, higher Movement Disorder Society modified Unified Parkinson's Disease Rating Scale (MDS‐UPDRS) part I–III, Hoehn and Yahr stages, and frequent falls might be associated with sarcopenia.^4^, ^5^ In contrast, it is also recognized that non‐motor symptoms (NMS) affect motor symptoms and the patient's quality of life (QOL).^4^, ^6^ Among the NMS, autonomic dysfunction is particularly important because of its high prevalence and poor prognosis in patients with PD.^7^ In healthy older adults and individuals with atypical Parkinson's syndrome, such as multiple system atrophy and progressive supranuclear palsy, autonomic dysfunction might be related to sarcopenia.^8^, ^9^ However, limited research has focused on the association between autonomic dysfunction and sarcopenia in patients with PD.

Therefore, the present study aimed to elucidate the relationship between autonomic dysfunction, assessed using the Scales for Outcomes in Parkinson's Disease‐Autonomic Questionnaire (SCOPA‐AUT), and the presence of sarcopenia. The relationship between each domain of the SCOPA‐AUT and sarcopenia was subsequently examined, and the test accuracy was evaluated by determining the optimal cut‐off.

## Methods

### Patient selection

Patients with PD were selected from the National Center of Neurology and Psychiatry PD registry, a prospective database of patients with PD admitted as part of our original hospitalization program for evaluation and rehabilitation.^10^ Patients with PD were retrospectively screened between March 2015 and February 2024.

The inclusion criteria were as follows: (1) patients diagnosed by well‐trained neurologists with clinically established or probable PD based on the Movement Disorder Society Clinical Diagnostic Criteria for PD^11^; (2) availability of data to assess sarcopenia; (3) availability of the SCOPA‐AUT; (4) patients with Hoehn and Yahr stages 1–3 at the on‐periods; and (5) the availability of patients' data at initial admission in the case of multiple admissions. The exclusion criteria were as follows: (1) apparent dementia with a Mini‐Mental State Examination score <24 or missing Mini‐Mental State Examination data^12^; (2) comorbid diseases affecting sarcopenia, such as active cancer, severe renal failure (estimated glomerular filtration rate [eGFR] <15 mL/min/1.73 m^2^ or undergoing renal replacement therapy), infectious disease or muscular disease^13^; and (3) contraindications to bioelectrical impedance analysis, such as implanted medical devices and deep brain stimulation.^8^

### Patients' clinical characteristics and evaluation for non‐motor symptoms

Patients' baseline data included sex; age; body mass index (BMI); disease duration; smoking history (current or past smoker); current alcohol consumption; hypertension; diabetes mellitus; polypharmacy, defined as five or more medications per day;^14^ the use of psychotropic drugs; the levodopa equivalent daily dose^15^, ^16^; constipation, defined as the use of laxatives or absence of daily bowel movements; orthostatic hypotension, defined as taking oral vasopressor drug or a decrease in systolic and/or diastolic blood pressure of ≥20 mmHg and/or ≥ 10 mmHg when transitioning from the supine to an upright position^8^; Hoehn and Yahr stage at on‐periods; MDS‐UPDRS parts I, II, III and IV scores; tests to evaluate for NMS of PD; laboratory data related to sarcopenia (prealbumin, retinol‐binding protein and transferrin); grip strength; appendicular skeletal mass index; bone mineral density at the lumbar vertebra and whole body; and positive cardiac ^123^I‐metaiodobenzylguanidine scintigraphy performed at or before hospital admission, defined as a heart‐to‐mediastinum ratio ≤2.2 in the delayed phase.^17^

The NMS in PD, including autonomic dysfunction, cognition, sleep disorders, mood and hyposmia, were assessed at admission in this study.^18^ The SCOPA‐AUT was used to predict the severity of autonomic dysfunction. The SCOPA‐AUT comprises 23 questions constituting six domains: gastrointestinal, urinary, cardiovascular, thermoregulatory, pupillomotor and sexual functions. Each question is scored from 0 (never) to 3 (often). Because of the lower number of valid responses regarding sexual function among Japanese persons, five domains were aggregated, excluding sexual function.^19^ For other NMS, cognitive evaluation included the Mini‐Mental State Examination, Frontal Assessment Battery and Japanese version of the Montreal Cognitive Assessment (MoCA‐J).^20^ The Epworth Sleepiness Scale, Pittsburgh Sleep Quality Index, Insomnia Severity Index and Rapid Eye Movement Sleep Behavior Disorder Screening Questionnaire Japanese version were used to evaluate subjective sleepiness, sleep quality, insomnia and rapid eye movement sleep behavior disorders.^21^, ^22^ Depression and anxiety were assessed using the Beck Depression Inventory and State–Trait Anxiety Inventory Japanese versions separated by Y‐1 and Y‐2 sections, reflecting state and trait anxiety. The symptoms of impulsive‐compulsive disorders were evaluated using the Questionnaire for Impulsive‐Compulsive Disorders in Parkinson's Disease. Hyposmia was defined as moderate to severe olfactory impairment with an average recognition threshold >2.5 on T&T olfactometry.^23^ The patients' habitual activity levels were also estimated using the Physical Activity Scale for the Elderly.

### Definition of sarcopenia

Sarcopenia was defined as decreased skeletal muscle mass and lower muscle strength.^24^ Skeletal muscle mass was evaluated using whole‐body dual‐energy X‐ray absorptiometry (2015–2017, Explorer DEXA Scanner; Hologic, Massachusetts, Marlborough, USA. 2017–, Horizon DEXA Scanner; Hologic, Massachusetts, Marlborough, USA) carried out by radiological technologists. Specifically, the former was defined as appendicular skeletal mass index <7.0 kg/m^2^ for men or <5.4 kg/m^2^ for women according to the criteria for Asian populations (The Asian Working Group for Sarcopenia).^24^ The appendicular skeletal mass index was calculated based on the following formula: appendicular skeletal muscle mass (fat‐free mass in the upper and lower extremities) divided by height squared. Handgrip strength was measured using a handheld dynamometer (Takei Scientific Instruments, Niigata, Japan). Measurements were obtained with the patient in a seated position and with the elbow extended. The latter was defined as the maximum grip strength of the dominant hand or both hands from multiple measurements, <28 kg for men and <18 kg for women, according to the Asian Working Group for Sarcopenia 2019.^24^

### Statistical analysis

Initially, baseline characteristics were compared between patients with and without sarcopenia using the χ^2^‐test, Fisher's exact test and the Mann–Whitney U‐test, as appropriate.

Subsequently, a Poisson regression model with a robust variance estimator was used to estimate the prevalence ratio (PR) and 95% confidence interval (CI) of variables associated with sarcopenia.^25^ The analysis included the following two models, because a large number of independent variables were estimated considering the small sample size of the study: model 1 was adjusted for age and BMI, which are well‐known independent risk factors for sarcopenia,^26^ along with each background factor with *P* < 0.05 in univariate analyses, resulting in multiple three‐variable models; and model 2 was adjusted for age, BMI and for background factors with *P* < 0.05 in model 1.

Furthermore, the specific domains of the SCOPA‐AUT that could be related to sarcopenia were evaluated using the same statistical methods for each domain, as described above.

Finally, receiver operating characteristic analysis was carried out to assess the predictive performance of SCOPA‐AUT for sarcopenia, as the optimal cut‐off value of SCOPA‐AUT had not been determined. The optimal cut‐off point was estimated based on the Youden index, because the cut‐off of the SCOPA‐AUT was not previously determined.

To avoid multicollinearity among the independent variables, variance inflation factors were examined. Only one variable was entered into the multivariable regression modeling procedure for variables that were highly correlated (variance inflation factor >10). Values of *P* < 0.05 were considered statistically significant for all results. All statistical analyses were carried out using Stata version 18 (StataCorp, College Station, TX, USA).

## Results

Consecutive patients (*n* = 255) admitted as part of our original hospitalization program were screened, of which 124 patients with PD were included (median age 68 years, 76 [61%] men). Of the 124 patients with PD, sarcopenia was identified in 31 (25%). The patient characteristics and each item of the SCOPA‐AUT were compared between patients with and without sarcopenia using univariate analysis (Table 1 and Table 2).

**Table 1:** Clinical characteristics of patients with Parkinson's disease with and without sarcopenia

	Total	With sarcopenia	Without sarcopenia	
Variable	(*n* = 124)	(*n* = 31)	(*n* = 93)	*P*‐value
Male sex	76 (61)	22 (71)	54 (58)	0.201
Age (years)	68 (62–74)	74 (68–78)	66 (60–70)	<0.001
Body mass index (kg/m^2^)	22.5 (20.1–25.1)	20.9 (18.9–22.9)	23.1 (20.6–25.7)	<0.001
Disease duration (years)	6 (4–10)	6 (3–10)	6 (4–10)	0.878
Smoking, *n* (%)	40 (32)	12 (39)	28 (30)	0.375
Alcohol intake, *n* (%)	24 (19)	8 (26)	16 (17)	0.307
Hypertension, *n* (%)	41 (33)	8 (26)	33 (35)	0.321
Diabetes mellitus, *n* (%)	5 (4)	1 (3)	4 (4)	0.792
Polypharmacy, *n* (%)	87 (70)	19 (61)	68 (73)	0.213
Psychotropic drug, *n* (%)	44 (35)	8 (26)	36 (39)	0.193
Levodopa equivalent daily dose (mg)	450 (300–635)	400 (300–600)	499 (300–650)	0.266
Constipation, *n* (%)	89 (72)	27 (87)	62 (67)	0.029
Orthostatic hypotension, *n* (%)	39 (31)	16 (52)	23 (25)	0.005
Hoehn & Yahr stage	2 (2–3)	2 (2–3)	2 (2–3)	0.587
MDS‐UPDRS part I	8 (5–12)	9 (7–12)	8 (5–11)	0.158
MDS‐UPDRS part II	13 (8–17)	16 (11–19)	12 (8–16)	0.005
MDS‐UPDRS part III	26 (18–35)	33 (25–37)	25 (17–34)	0.017
MDS‐UPDRS part IV	0 (0–4)	0 (0–3)	0 (0–5)	0.663
Non‐motor symptoms				
SCOPA‐AUT	12 (8–17)	18 (12–23)	11 (8–15)	<0.001
MMSE	29 (27–30)	29 (27–30)	29 (28–30)	0.611
FAB	16 (15–18)	15 (14–17)	16 (15–18)	0.048
MoCA‐J	25 (23–27)	25 (22–28)	25 (23–27)	0.758
ESS	9 (6–13)	8 (6–14)	9 (6–13)	0.991
PSQI	8 (5–11)	8 (5–10)	8 (6–11)	0.691
ISI	8 (5–14)	7 (3–14)	8 (6–14)	0.412
RBDSQ‐J	4 (2–6)	4 (1–7)	4 (2–6)	0.547
BDI	14 (9–19)	15 (11–19)	13 (7–19)	0.193
STAI‐Y1	45 (38–51)	46 (39–52)	44 (38–50)	0.678
STAI‐Y2	47 (39–53)	48 (43–53)	46 (37–53)	0.269
QUIP	0 (0–1)	0 (0–0)	0 (0–1)	0.629
Hyposmia	60 (48)	18 (58)	42 (45)	0.252
PASE	75.4 (45.2–110.3)	56.2 (40.1–98.6)	81.4 (48.7–111.0)	0.134
Laboratory data				
Prealbumin (mg/dL)	25.1 (22.7–29.2)	23.2 (20.9–26.5)	25.6 (23.8–29.7)	0.005
Retinol‐binding protein (mg/dL)	3.1 (2.7–3.4)	2.9 (2.6–3.2)	3.2 (2.7–3.6)	0.025
Transferrin (mg/dL)	229 (212–257)	226 (194–250)	230 (218–257)	0.116
Grip hand (kg)	26.8 (19.5–32.8)	22.1 (15.0–26.8)	29.9 (21.0–35.0)	<0.001
Appendicular skeletal mass index (kg/m^2^)	6.39 (5.80–7.16)	5.94 (5.21–6.43)	6.54 (5.89–7.37)	<0.001
BMD at lumbar vertebra (%)	84 (73–98)	77 (68–96)	88 (75–99)	0.046
BMD at whole body (%)	91 (84–99)	86 (81–95)	91 (85–100)	0.062
Positive MIBG, *n* (%)	87 (70)	21 (68)	66 (71)	0.304

*Note*: Data are presented as the median (interquartile range) or number (%). BDI, Beck Depression Inventory second edition; BMD, bone mineral density; ESS, Epworth Sleepiness Scale; FAB, Frontal Assessment Battery; ISI, Insomnia Severity Index; MDS‐UPDRS, Movement Disorder Society Modified Unified Parkinson's Disease Rating Scale; MIBG, cardiac ^123^I‐metaiodobenzylguanidine scintigraphy, MMSE, Mini‐Mental State Examination; MoCA‐J, Japanese version of the Montreal Cognitive Assessment; PASE, Physical Activity Scale for the Elderly; PSQI, Pittsburgh Sleep Quality Index; QUIP, Questionnaire for Impulsive‐Compulsive Disorders in Parkinson's disease; RBDSQ‐J, Rapid Eye Movement Sleep Behavior Disorder Screening Questionnaire Japanese version; SCOPA‐AUT, Scales for Outcomes in Parkinson's Disease‐Autonomic Questionnaire; STAI, State–Trait Anxiety Inventory.

**Table 2:** Comparison of Scales for Outcomes in Parkinson's Disease‐Autonomic Questionnaire scores between Parkinson's disease patients with and without sarcopenia

	Total	With sarcopenia	Without sarcopenia	
Variable	(*n* = 124)	(*n* = 31)	(*n* = 93)	*P*‐value
Gastrointestinal function	4 (2–7)	8 (5–9)	3 (2–6)	<0.001
1. Swallowing/choking	0 (0–1)	1 (0–1)	0 (0–1)	0.045
2. Sialorrhea	1 (0–1)	1 (1–2)	0 (0–1)	0.001
3. Dysphagia	0 (0–1)	0 (0–1)	0 (0–0)	0.013
4. Early abdominal fullness	0 (0–1)	1 (0–1)	0 (0–1)	0.062
5. Constipation	1 (0–2)	1 (1–2)	1 (0–2)	0.016
6. Straining for defecation	1 (0–2)	2 (1–3)	1 (0–2)	0.020
7. Fecal incontinence	0 (0–0)	0 (0–1)	0 (0–0)	0.007
Urinary function	5 (4–8)	6 (4–10)	5 (3–6)	0.030
8. Urinary urgency	1 (0–1)	1 (0–1)	0 (0–1)	0.469
9. Urinary incontinence	0 (0–1)	1 (0–1)	0 (0–1)	0.154
10. Incomplete emptying	0 (0–1)	0 (0–1)	0 (0–1)	0.340
11. Weak stream of urine	1 (0–1)	1 (0–2)	0 (0–1)	0.037
12. Frequency	1 (1–2)	1 (1–3)	1 (1–2)	0.206
13. Nocturia	2 (1–3)	2 (2–3)	2 (1–3)	0.013
Cardiovascular function	0 (0–1)	1 (0–2)	0 (0–1)	0.022
14. Lightheaded (standing up)	0 (0–1)	1 (0–1)	0 (0–1)	0.028
15. Lightheaded (standing some time)	0 (0–0)	0 (0–1)	0 (0–0)	0.248
16. Syncope	0 (0–0)	0 (0–0)	0 (0–0)	0.793
Thermoregulatory function	1 (0–2)	1 (0–3)	1 (0–2)	0.759
17. Hyperhidrosis during the day	0 (0–0)	0 (0–0)	0 (0–0)	0.763
18. Hyperhidrosis during the night	0 (0–1)	0 (0–1)	0 (0–1)	0.763
20. Cold intolerance	0 (0–0)	0 (0–1)	0 (0–0)	0.149
21. Heat intolerance	0 (0–0)	0 (0–0)	0 (0–1)	0.429
Pupillomotor function	0 (0–1)	0 (0–1)	0 (0–1)	0.018
19. Oversensitive to bright light	0 (0–1)	0 (0–1)	0 (0–1)	0.018

*Note*: Data are presented as the median (interquartile range).

Abbreviations: SCOPA‐AUT, Scales for Outcomes in Parkinson's Disease‐Autonomic Questionnaire.

In the Poisson regression analysis with the robust variance estimator of model 1, three factors were independently and significantly associated with sarcopenia: MDS‐UPDRS Part II (PR 1.075, 95% CI 1.036–1.116, *P* < 0.001), MDS‐UPDRS Part III (PR 1.027, 95% CI 1.000–1.054, *P* = 0.047) and SCOPA‐AUT (PR 1.078, 95% CI 1.034–1.122, *P* < 0.001; Table 3). In model 2, adjusted for age, BMI, SCOPA‐AUT, and MDS‐UPDRS Part II and Part III, it was confirmed that a higher SCOPA‐AUT score might be associated with sarcopenia (PR 1.069, 95% CI 1.024–1.116; *P* < 0.001; Table 3).

**Table 3:** Poisson regression analysis with a robust variance estimator of clinical factors and Scales for Outcomes in Parkinson's Disease‐Autonomic Questionnaire domains associated with sarcopenia in patients with Parkinson's disease

	Crude			Model 1			Model 2		
	PR	95% CI	*P*‐value	PR	95% CI	*P*‐value	PR	95% CI	*P*‐value
Clinical factors									
Age	1.086	1.052–1.121	<0.001				1.081	1.034–1.123	<0.001
Body mass index	0.825	0.757–0.898	<0.001				0.860	0.803–0.922	<0.001
MDS‐UPDRS part II	1.068	1.033–1.105	<0.001	1.075	1.036–1.116	<0.001	1.044	0.997–1.094	0.068
MDS‐UPDRS part III	1.029	1.005–1.053	0.015	1.027	1.000–1.054	0.047	1.019	0.992–1.047	0.160
SCOPA‐AUT	1.085	1.040–1.131	<0.001	1.078	1.034–1.122	<0.001	1.069	1.024–1.116	<0.001
Constipation	2.654	0.998–7.062	0.051	2.171	0.912–5.168	0.080			
Orthostatic hypotension	2.461	1.295–4.677	0.006	1.388	0.716–2.690	0.331			
FAB	0.881	0.820–0.946	0.001	0.903	0.805–1.012	0.080			
Prealbumin	0.922	0.874–0.972	0.003	0.976	0.912–5.168	0.080			
Retinol‐binding protein	0.596	0.395–0.900	0.014	0.863	0.574–1.296	0.477			
BMD at lumbar vertebra	0.983	0.965–1.002	0.086	1.002	0.984–1.019	0.839			
SCOPA‐AUT domains									
Gastrointestinal functioning	1.223	1.114–1.343	<0.001	1.156	1.045–1.277	0.005	1.131	1.006–1.272	0.039
Urinary functioning	1.106	1.044–1.172	0.001	1.103	1.053–1.156	<0.001	1.103	1.037–1.172	0.002
Cardiovascular functioning	1.127	0.942–1.349	0.191	1.115	0.939–1.324	0.213	1.117	0.919–1.358	0.266
Thermoregulatory functioning	1.024	0.878–1.194	0.766	1.079	0.948–1.229	0.248	1.079	0.936–1.243	0.296
Pupillomotor functioning	1.673	1.288–2.173	<0.001	1.461	1.164–1.833	0.001	1.480	1.127–1.942	0.014

*Note*: Model 1: A Poisson regression model with a robust variance estimator was adjusted for age and body mass index, along with each background factor statically significant in univariate analyses (*P* < 0.05; MDS‐UPDRS part II, MDS‐UPDRS part III, SCOPA‐AUT, constipation, orthostatic hypotension, FAB, prealbumin, retinol‐binding protein and BMD at the lumbar vertebra), resulting in multiple three‐variable models. Model 2: A Poisson regression model with a robust variance estimator was adjusted for age, body mass index and background factors (*P* < 0.05 in model 1 (MDS‐UPDRS Part II, MDS‐UPDRS Part III and SCOPA‐AUT). The specific domains of the SCOPA‐AUT that could be related to sarcopenia were evaluated using the same statistical methods as in model 1 and 2. Only the results for each domain of SCOPA‐AUT are presented.

Abbreviations: BMD, bone mineral density; CI, confidence interval; FAB, frontal Assessment Battery; MDS‐UPDRS, Movement Disorder Society Modified Unified Parkinson's Disease Rating Scale; PR, prevalence ratio; SCOPA‐AUT, Scales for Outcomes in Parkinson's Disease‐Autonomic Questionnaire.

Subsequently, Poisson regression analysis with a robust variance estimator in each domain of the SCOPA‐AUT showed that higher scores for gastrointestinal, urinary and pupillomotor functioning in the SCOPA‐AUT were significantly associated with sarcopenia (all *P* < 0.05 in models 1 and 2; Table 3).

**Figure 1:**
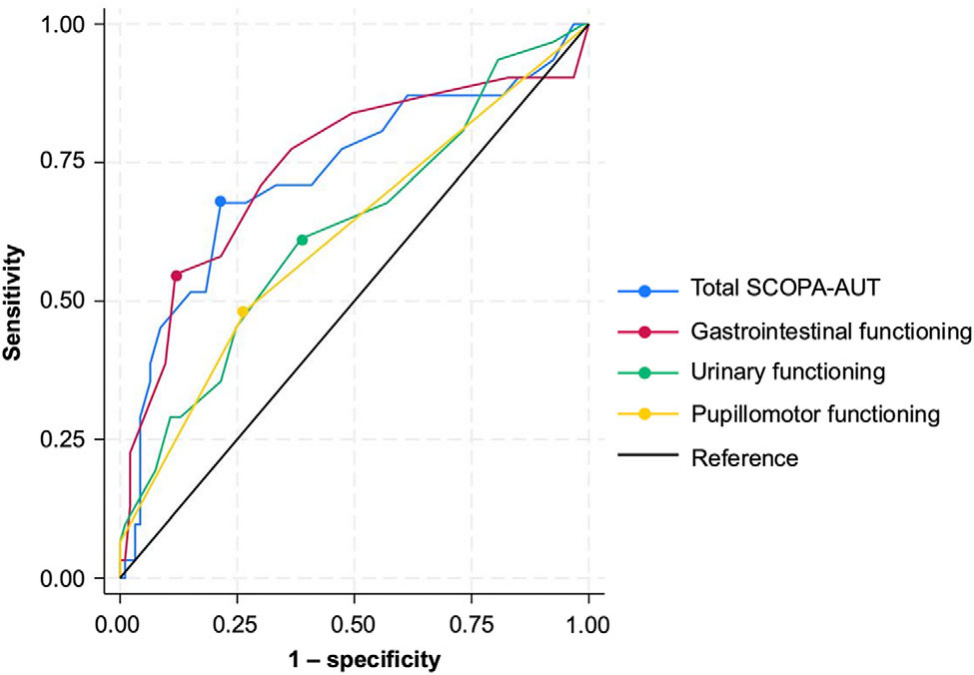
Receiver operating characteristic analysis of total Scales for Outcomes in Parkinson's Disease‐Autonomic Questionnaire (SCOPA‐AUT) and each statistically significant domain to distinguish sarcopenia. The optimal cut‐off point is estimated based on the Youden index.

Finally, receiver operating characteristic analysis showed that SCOPA‐AUT is a reliable predictor of sarcopenia, with an area under the curve (AUC) of 0.730 (95% CI 0.615–0.844), sensitivity (Se) of 0.677 and specificity (Sp) of 0.785 at a SCOPA‐AUT cut‐off of 16 (Figure 1 and Table 4). In each domain of the SCOPA‐AUT, gastrointestinal function (AUC 0.744, Se 0.548, Sp 0.882, cut‐off 8) was a more reliable predictor of sarcopenia than urinary function (AUC 0.629, Se 0.613, Sp 0.613, cut‐off 6) and pupillomotor function (AUC 0.616, Se 0.484, Sp 0.731, cut‐off 1; Figure 1 and Table 4).

**Table 4:** Accuracy for total Scales for Outcomes in Parkinson's Disease‐Autonomic Questionnaire score and each domain for diagnosis of sarcopenia in patients with Parkinson's disease

	With sarcopenia, *n* (%)	Without sarcopenia, *n* (%)	Se	Sp	Cut‐off value	AUC	95% CI	*P*‐value
Total	21 (68)	20 (21)	0.677	0.785	16	0.730	0.615–0.844	<0.001
Gastrointestinal functioning	17 (55)	11 (12)	0.548	0.882	8	0.744	0.630–0.858	<0.001
Urinary functioning	19 (61)	36 (39)	0.613	0.613	6	0.629	0.512–0.747	0.028
Pupillomotor functioning	15 (48)	25 (27)	0.484	0.731	1	0.616	0.513–0.719	0.027

Abbreviations: AUC, area under the curve; CI, confidence interval; SCOPA‐AUT, Scales for Outcomes in Parkinson's Disease‐Autonomic Questionnaire; Se, sensitivity; Sp, specificity.

## Discussion

The present study showed the following significant findings: (1) a higher SCOPA‐AUT score could predict the presence of sarcopenia in patients with PD; (2) a higher score of SCOPA‐AUT, particularly in the domains of gastrointestinal, urinary and pupillomotor functioning, could be significantly associated with the presence of sarcopenia; and (3) the total score of SCOPA‐AUT and the score of the gastrointestinal functioning domain might be reliable predictive tools for sarcopenia, as indicated by receiver operating characteristic analysis. Although determining the objective severity of autonomic dysfunction involves an arduous process, self‐questionnaires using the SCOPA‐AUT can be simple and effective in predicting the presence of sarcopenia in patients with PD.

Previous studies have suggested that longer disease duration, severe motor impairment, frequent falls and the burden of NMS are associated with sarcopenia.^4^, ^5^ Indeed, the prognosis and QOL for patients with PD might be affected not only by motor symptoms, but also by NMS, which has recently been recognized as a more frequent and important factor.^6^, ^27^ Evaluating NMS tends to be challenging, because patients with PD might be reluctant to mention NMS, so questionnaires can be useful to assist in evaluating NMS.^6^ However, few studies have focused on the association between NMS and sarcopenia, and have shown that poor sleep quality and fatigue might be associated with sarcopenia.^26^ Notably, this study included a large number of relatively early‐stage and active patients with PD, and illustrated that the subjective severity of autonomic dysfunction is associated with sarcopenia using various types of questionnaires for NMS. These findings highlight the importance of autonomic dysfunction in the development of sarcopenia in patients with PD.

There are a few reports on the exact mechanism underlying the association between autonomic dysfunction and sarcopenia in patients with PD; however, one possibility might involve background inflammation. Chronic inflammation plays a significant role in the onset and progression of sarcopenia.^28^ Recently, it has been reported that PD is associated with a predisposition for neuroinflammation.^29^ It has been suggested that patients with PD with severe autonomic symptoms, particularly enteric symptoms, tend to trigger background neuroinflammation via the gut–vagus–brain signaling.^30^, ^31^, ^32^ In animal models of PD, mice with intestinal autonomic dysfunction showed the accumulation of phosphorylated α‐synuclein combining Toll‐like receptor 2.^33^ This process induces the Toll‐like receptor 2/nuclear factor kappa‐B signaling pathway, thereby activating neuroinflammatory responses.^34^ Therefore, the aforementioned theory could support the present finding that a high SCOPA‐AUT score for the gastrointestinal functioning domain is related to sarcopenia due to severe background neuroinflammation.

Regarding the specific items of the SCOPA‐AUT, elevated scores for swallowing/choking, sialorrhea and dysphagia suggest that subjective dyspepsia might lead to decreased calorie intake (Table 2).^2^ Gastrointestinal dysmotility, such as delayed gastric emptying and constipation, can interfere with optimal bioavailability of levodopa, impair nutrient absorption and reduce overall dietary intake, leading to malnutrition and muscle wasting.^35^ A previous report also showed that patients with PD and severe autonomic disturbances might experience poor QOL and fatigue.^36^ In the present study, symptoms of urinary tract and pupil abnormalities could impair the QOL in patients with PD.^37^, ^38^ These symptoms could result in discomfort, frustration and difficulty managing daily activities, which can make patients hesitant to engage in physical activities. As a result, they tend to show reduced levels of physical activity, leading to muscle loss. Therefore, patients with PD with severe autonomic disturbances are more likely to develop sarcopenia because of their low‐calorie intake and limited physical activity.^39^

From the perspective of dopaminergic medications, the relationship between dopaminergic therapy and both autonomic dysfunction and sarcopenia is likely to be complex and context‐dependent. Although dopaminergic medications might improve physical activity and motor function by alleviating core motor symptoms,^40^ they might also exert unintended metabolic effects or worsen certain autonomic symptoms, such as delayed gastric emptying and orthostatic hypotension.^35^, ^41^ Conversely, advanced formulations, such as levodopa‐carbidopa intestinal gel, might provide more stable systemic effects, potentially supporting autonomic stability.^42^ Thus, L‐dopa therapy alone might not be sufficient to simultaneously manage autonomic dysfunction and sarcopenia. A multidisciplinary strategy combining pharmacological optimization, autonomic symptom‐specific interventions, nutritional support and tailored physical rehabilitation might be necessary. Future studies are warranted to investigate the interactive effects of these approaches.

These findings have important clinical implications, necessitating consideration of the influence of autonomic dysfunction on the presence of sarcopenia in patients with relatively active PD without apparent dementia. As sarcopenia itself is strongly associated with the progression of PD and the frequency of falls,^4^, ^5^ it is important to recognize the significance of clarifying and controlling the risk factors for sarcopenia. Given that the SCOPA‐AUT is a straightforward and cost‐effective test, it should be routinely administered at the time of hospital admission or outpatient visits to identify high‐risk patients with PD who might develop sarcopenia. For screening‐positive patients with PD, a short‐term hospitalization might be beneficial for carrying out multidisciplinary assessments, including videoendoscopic or videofluoroscopic evaluation for dysphagia, detailed testing and medical intervention for autonomic symptoms, and nutritional intervention by a support team, which might help prevent sarcopenia progression. Furthermore, determining a causal relationship between whether autonomic dysfunction or sarcopenia comes first remains challenging, akin to the “chicken and egg” dilemma. However, we postulate that autonomic dysfunction and sarcopenia might interact in a bidirectional manner, potentially forming a vicious cycle that contributes to poor prognosis in patients with PD.^43^ In the future, a prospective study to clarify whether early and aggressive interventions for autonomic dysfunction can prevent or ameliorate the progression of sarcopenia in patients with PD on longitudinal follow up would be of interest. Exercise regimens aimed at improving sarcopenia might also have a beneficial impact on autonomic symptoms,^43^, ^44^ potentially resulting in a positive feedback loop where improvement in sarcopenia could also alleviate autonomic symptoms interactively.

A major strength of this study was the inclusion of relatively active patients with PD without apparent dementia, which enabled a rigorous and comprehensive assessment of non‐motor symptoms using various standardized batteries, ultimately identifying autonomic dysfunction as the most influential factor associated with sarcopenia. However, the present study had some limitations. First, the actual associations between autonomic dysfunction and background neuroinflammation were not examined. Future studies exploring the association between neuroinflammatory markers—such as interleukin‐6 and tumor necrosis factor‐α in serum or cerebrospinal fluid—and autonomic dysfunction might provide deeper insights into the pathophysiological mechanisms underlying our findings. Furthermore, self‐reported subjective accuracy by SCOPA‐AUT might be influenced by cognitive fluctuations, even in patients with PD with relatively preserved cognitive function, like the cohort in this study. Second, the objective assessment of autonomic dysfunction in this study was limited. A more comprehensive evaluation of autonomic function, incorporating objective measures, would have strengthened our findings based primarily on subjective assessments. Third, genetic tests were not carried out on all patients with PD. Only one patient with mutations in the glucocerebrosidase gene was included in the present study cohort. Fourth, the actual gait speed was not examined. Gait speed is required for judging severe sarcopenia based on the Asian Working Group for Sarcopenia 2019^24^; but this study used appendicular skeletal mass index and grip strength, because gait speed might be greatly influenced by freezing of gait and off‐time in patients with PD. Furthermore, as this was a retrospective study, gait speed was not routinely assessed in our clinical practice, limiting the availability of gait speed data. Thus, the lack of gait speed assessment could affect the diagnostic accuracy of sarcopenia. Finally, the inclusion of only East Asian patients from a single hospital center might have compromised the internal and external validity of the study. The difference in BMI between Asian and Western patients might limit the generalizability of the study findings.^45^

In summary, a higher SCOPA‐AUT score, particularly in the gastrointestinal function domain, could serve as a viable predictive marker for sarcopenia in patients with PD.

## Disclosure statement

Motohiro Okumura, Reika Saika and Yuji Takahshi declare no conflict of interest. Yohei Mukai has received financial compensation for lectures and scientific consultations from AbbVie, not relevant to this work.

## Author contributions

All authors contributed sufficiently to the manuscript to be included as authors.

M. O.: Conceptualization, Data curation, Formal analysis, Investigation, Methodology, Project administration, Resources, Software, Validation, Visualization, Writing–original draft. Y. M.: Conceptualization, Funding acquisition, Methodology, Project administration, Resources, Software, Supervision, Validation, Writing–review and editing. R. S.: Project administration, Resources, Supervision, Writing–review and editing. Y. T.: Project administration, Resources, Supervision, Writing–review and editing.

All authors read and approved the final manuscript.

## Ethics statement

This clinical study was approved by the Ethics Committee of the National Center of Neurology and Psychiatry (approval number A2010‐014), and conducted in accordance with the Declaration of Helsinki. Information regarding this study was published on the National Center of Neurology and Psychiatry website. Written informed consent was obtained from all patients. We confirm that we have read the Journal's position on issues involved in ethical publication and affirm that this work is consistent with those guidelines.

## Data Availability

The data that support the findings of this study are available on request from the corresponding author. The data are not publicly available due to privacy or ethical restrictions.
